# ACE2 and Innate Immunity in the Regulation of SARS-CoV-2-Induced Acute Lung Injury: A Review

**DOI:** 10.3390/ijms222111483

**Published:** 2021-10-25

**Authors:** Lihua Qu, Chao Chen, Tong Yin, Qian Fang, Zizhan Hong, Rui Zhou, Hongbin Tang, Huifen Dong

**Affiliations:** 1 Department of Pathogenic Biology, School of Basic Medical Sciences, Wuhan University, Wuhan 430071, China; lihuaqu@whu.edu.cn (L.Q.); yintong@whu.edu.cn (T.Y.); qianfang@whu.edu.cn (Q.F.); zizhanhong@whu.edu.cn (Z.H.); ruizhou@whu.edu.cn (R.Z.); 2 School of Medicine & Holistic Integrative Medicine, Nanjing University of Chinese Medicine, Nanjing 210013, China; 20193068@njucm.edu.cn; 3 Center for Animal Experiment, State Key Laboratory of Virology, Wuhan University, Wuhan 430071, China

**Keywords:** ACE2, immune cells, COVID-19, SARS-CoV-2, ALI

## Abstract

Despite the protracted battle against coronavirus acute respiratory infection (COVID-19) and the rapid evolution of the severe acute respiratory syndrome coronavirus 2 (SARS-CoV-2), no specific and effective drugs have to date been reported. Angiotensin-converting enzyme 2 (ACE2) is a zinc metalloproteinase and a critical modulator of the renin-angiotensin system (RAS). In addition, ACE2 has anti-inflammatory and antifibrosis functions. ACE has become widely known in the past decade as it has been identified as the primary receptor for SARS-CoV and SARS-CoV-2, being closely associated with their infection. SARS-CoV-2 primarily targets the lung, which induces a cytokine storm by infecting alveolar cells, resulting in tissue damage and eventually severe acute respiratory syndrome. In the lung, innate immunity acts as a critical line of defense against pathogens, including SARS-CoV-2. This review aims to summarize the regulation of ACE2, and lung host cells resist SARS-CoV-2 invasion by activating innate immunity response. Finally, we discuss ACE2 as a therapeutic target, providing reference and enlightenment for the clinical treatment of COVID-19.

## 1. Introduction

Coronavirus acute respiratory infection 2019 (COVID-19) induced by severe acute respiratory syndrome coronavirus 2 (SARS-CoV-2) is a worldwide acute respiratory disease. SARS-CoV-2 has higher infectivity than Middle East respiratory syndrome coronavirus (MERS-CoV) and severe acute respiratory syndrome coronavirus (SARS-CoV) [1,2,3]. The pathological features of COVID-19 are similar to SARS and MERS, including extensive edema, hyaline membrane formation, inflammatory infiltration, micro-thrombosis, and fibrosis [4,5]. Currently, there are more than 185 million COVID-19 cases worldwide, 10–20% of which have manifested acute lung injury (ALI) and even developed acute respiratory distress syndrome (ARDS), with an associated mortality of about 3% [1,6,7,8]. ALI is characterized by increased permeability of the pulmonary capillary and numerous immune cells, such as macrophages, neutrophils, lymphocytes, and dendritic cells (DCs), participate in the inflammatory response against SARS-CoV-2. A potent response against the virus can induce a cytokine storm and lead to ALI [9,10,11,12]. 

Angiotensin-converting enzyme 2 (ACE2) is a homolog of angiotensin-converting enzyme (ACE) expressed in human lungs and immune cells. ACE2 is known to antagonize the renin-angiotensin system (RAS) to alleviate ALI [13]. After the SARS outbreak in 2003, the critical role of ACE2 in ALI/ARDS has attracted widespread attention. Clinical studies showed that the insertion or deletion of ACE could impact the severity of ARDS [14,15]. Studies have reported that SARS-CoV2 has highly homologous sequences with SARS-CoV, which binds to ACE2, causing ALI by using spikes [16,17]. ACE2 and pattern recognition receptors are molecules expressed by innate immune cells. This review discusses the role of ACE2 and innate immunity in SARS-CoV2, which provide novel therapies for the prevention and control of SARS-CoV-2.

## 2. ACE2 and COVID-19

### 2.1. The Biological Function of ACE2

**Figure 1:**
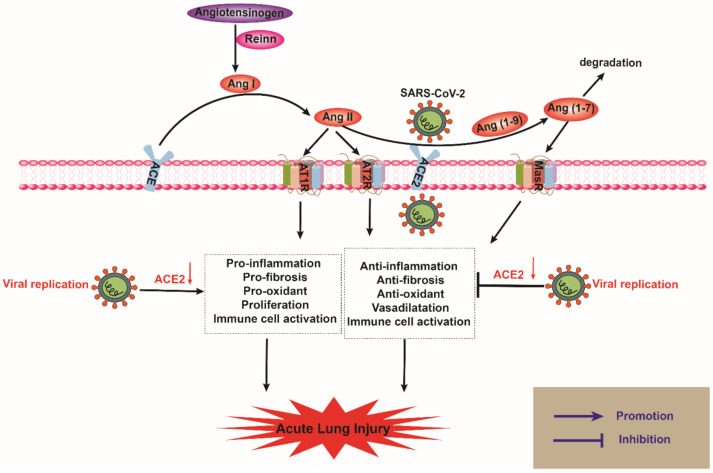
Current view on the renin angiotensin system (RAS) and development of ALI in COVID-19. RAS consists of classical and non-classical axes. ACE is a component of the classical axis, which converts angiotensin I (Ang-I) to Ang-II, and the latter binds to AT1 or AT2 receptors. The non-classical RAS axis contains ACE2, which hydrolyzes Ang II to produce Ang-(1-7). Ang-(1-7) has affinity with the Mas receptor (MasR), and they altogether form the ACE2/Ang-(1-7)/MasR axis, which can regulate the ACE/Ang II/AT1R axis. When lung tissues are infected with SARS-CoV-2, the virus binds with ACE2 receptor on the cell membrane and downregulates its expression, causing Ang II to activate the AT1 receptor and promote ALI. The red arrow indicates a reduction.

ACE2, and ACE homolog, belongs to the zinc metalloprotease family [18,19,20]. The *ACE2* gene is localized on the X chromosome with 18 exons, and it encodes a type I transmembrane glycoprotein composed of 805 amino acids, which includes an extracellular catalytic domain at the N-terminus and an intracellular sequence of 42 amino acids at the C-terminus [21,22]. The active site of the catalytic domain, the zinc metallopeptidase domain, shares 41.8% sequence identity with ACE. Although, there is some similarity, they catalyze different substrates and have different biological functions [23,24]. RAS is one of the essential vasoactive systems and plays a critical role in maintaining blood pressure and fluid–electrolyte balance through endocrine, paracrine, and autocrine systems [25]. In addition, it has been reported that RAS is involved in many inflammation-related pathological reactions [26,27,28]. RAS consists of several functionally interacting protease–hormone-receptor axes [29,30,31]. Among them, the ACE/angiotensin II (Ang II)/angiotensin type 1 receptor (AT1R) pathway is associated with cardiovascular fibrosis, oxidative stress, inflammation, cell apoptosis, proliferation, and cellular immunity [32,33]. ACE2 is a central player in the ACE2/Ang (1-9)/AT2R and ACE2/Ang- (1-7)/MasR axes [34]. ACE2 catalyzes the hydrolysis of angiotensin I (Ang I) to the nonapeptide Ang (1-9) and Ang II to the heptapeptide Ang (1-7), respectively [35]. Ang (1-9) and Ang (1-7) mainly bind to their corresponding G-protein-coupled receptors, AT2R, or Mas receptor (MasR), and exert biological effects of vasodilation, antifibrosis, anti-inflammatory, and immune cell activation [36,37] (Figure 1). ACE2 has a higher affinity for Ang II than Ang I, and the former works as its primary substrate [38,39,40].

### 2.2. ACE2 and SARS-CoV-2 in ALI

Audrey et al. [41] explored mice as a virus infection model, and found that SARS-CoV-2 could not replicate in wild-type mice. However, severe ALI occurred in mice by adapting the virus to mouse ACE2 receptor and inserting the human ACE2 gene. Zhou et al. [42] found that coronavirus entered the host cells by utilizing the spike protein to bind the hydrophobic pocket of the ACE2 extracellular catalytic domain. Once infected by the virus, ACE2 was downregulated in the cell and thus led to a dysregulation of the ACE2/Ang- (1-7)/MasR axis and the ACE/Ang II/AT1R axis [43]. Consequently, Ang II was upregulated, and AT1R was overstimulated, increasing capillary permeability, and causing pulmonary edema and ALI [44]. Imai et al. [23] demonstrated that the ACE2/Ang (1-7)/MasR axis has protective properties in the lungs, relieving pulmonary inflammation and fibrosis and inhibiting cancer cell growth, tumor angiogenesis, and metastasis. ACE2 also inhibited pulmonary fibrosis and lung injury caused by overactivation of the RAS system in ALI mediated by SARS and influenza viruses [44,45]. On the other hand, RAS inhibitors could upregulate ACE2 and reduce lung injury [23,46]. For instance, ACE2 knockout mice following treatment with an AT1R inhibitor or recombinant human ACE2 manifested less coronavirus-induced ALI [47,48]. Chen et al.’s [49] studies showed that glycyrrhizic acid attenuated inflammatory factors and adhesion molecules by upregulating ACE2 and inhibiting the caveolin-1/NF-κB signaling pathway, thereby alleviating LPS-induced ALI. Similarly, research on the influenza virus suggested that ACE2 is significantly downregulated upon H1N1 infection [50]. ACE2 deficiency deteriorated the condition of infected mice significantly, and AT1R blockade alleviated the lung damage caused by the H7N9 influenza virus [51,52]. In addition, an elevated level of Ang-II was found in patients with H5N1 or H7N9 infection, and these elevated levels were related to the severity of the pulmonary injury [52].

Gerard et al. [53] investigated the expression and distribution of ACE2 in COVID-19 and showed that the expression of ACE2 increased in lung tissues, serum, and endothelial cells but decreased in alveolar epithelial cells (AT2). Asaka et al. [54] explored the pathogenicity of COVID-19 and found that CAG-hACE2 mice were susceptible to SARS-CoV-2 and the levels of cytokines and chemokines increased, resulting in acute lung injury, while injecting the plasma of immunized mice could reduce the mortality of mice. Recent studies reported that SARS-CoV-2 infection contributed to RAS dysregulation [55,56]. SARS-CoV-2 patients had a higher level of Ang II, and administration of ACE inhibitors (ACEIs) could significantly reduce cytokine production and pulmonary inflammatory responses [57,58]. Therefore, drugs balancing the RAS axis, such as ACEIs and angiotensin receptor blockers, might be suitable for treating patients with COVID-19 [59,60,61]. In addition, previous studies reported coronavirus E protein as the determinant of SARS-CoV pathogenicity [62], as E protein triggered overexpression of inflammatory cytokines and aggravated the immune response, resulting in ALI, and eventually ARDS [61,63,64]. Collectively, these results suggested that suppression of RAS and ACE2 was involved in the pathogenesis of COVID-19 lung injury.

## 3. The Role of ACE2 in Innate Immune-Related Cells during SARS-CoV-2 Infection

ACE2 is abundantly expressed in alveolar epithelial cells, endothelial cells, macrophages, neutrophils, DCs, and lymphocytes in lung tissue [65,66,67]. Therefore, lung tissues are especially vulnerable to SARS-CoV2 invasion and the main target organ for virus attack [68,69,70].

SARS-CoV-2 binds to ACE2 in type II alveolar cells after entering the respiratory tract, resulting in decreased ACE2 and increased blood Ang II [71]. The association of Ang II with AT1R could induce bronchial smooth muscle contraction, pulmonary vascular hyperpermeability, alveolar epithelial cell apoptosis, and the release of numerous inflammatory cytokines and chemokines, which led to ARDS [72,73]. ACE2 on alveolar macrophages could relieve lung tissue injury and inflammation through adenosine monophosphate-activated protein kinase (AMPK) and mammalian target of rapamycin (mTOR) pathways [74,75,76]. Besides, the ACE2/Ang- (1-7)/MasR axis reduced the secretion of proinflammatory factors and apoptosis of alveolar epithelial cells and vascular endothelial cells by inhibiting JNK/NF-κB activation [77]. However, blockage of the MasR pathway impaired the protective effect of Ang- (1-7) and aggravated ARDS [78].

**Figure 2:**
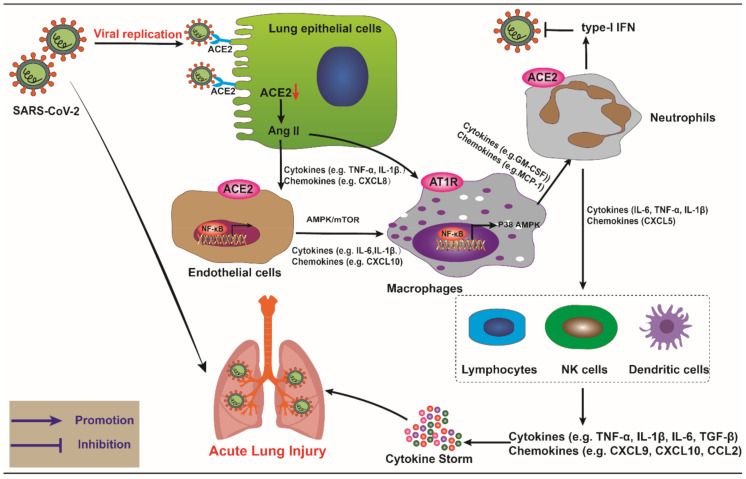
The cellular immune response of ALI induced by SARS-CoV2 infection. SARS-CoV-2 binds to ACE2 in type II alveolar cells after entering the respiratory tract, resulting in decreased ACE2 and increased Ang II. The association of Ang II with AT1R could induce bronchial smooth muscle contraction, pulmonary vascular hyperpermeability, alveolar epithelial release of numerous inflammatory cytokines, and chemokines. Alveolar macrophages could relieve lung tissue injury and inflammation through adenosine monophosphate-activated protein kinase (AMPK) and mammalian target of rapamycin (mTOR) pathways. Numerous cytokines and chemokines activate neutrophils to produce type-I IFN, inhibit virus infection, and neutrophils release numerous inflammatory factors and chemokines, promote the maturation of natural killer cells (NKs), dendritic cells (DCs), and the activation and migration of lymphocytes, activate the adaptive immune system, leading to further expansion of the immune response, and ultimately triggering a cytokine storm. The red arrow indicates a reduction.

Numerous immune cells are activated upon SARS-CoV-2 infection in the alveoli, which downregulates ACE2 and increases the expression of Ang II, proinflammatory IL-1β, IL-6, TNF-α, and the chemokines CXCL8, CXCL10 [79,80]. Ang II activates NF-κB and p38MAPK through the combination of AT1R, which produces numerous inflammatory factors, and in turn, activates the inflammatory response and triggers a massive accumulation of macrophages and neutrophils, aggravating lung injury [81]. Neutrophils, macrophages, and other immune cells are recruited to the lungs in the early stage of the disease to participate in the clearance of the virus [82]. Furthermore, viruses have a dynamic mutual interaction with alveolar epithelial cells and macrophages. The viruses induce the release of cytokines, such as monocyte chemotactic factor (MCP-1) and granulocyte-macrophage colony-stimulating factor (GM-CSF), by macrophages and neutrophils. Cytokine release activates macrophages and generates inflammatory factors, leading to lung inflammation and increased exudation. Alveolar expansion is also restricted, affecting the alveolar ventilation function, and eventually causing refractory hypoxemia and aggravating ALI [83,84]. Macrophages and neutrophils release IL-6, TNF-α, IL-1β, and CXCL5 to promote DCs’ maturation and the activation and migration of CD4+ and CD8+ T cells, which creates a microenvironment that is advantageous for immune cell migration and accumulation, activating the adaptive immune system [85]. In DCs, Ang-(1-7) stimulated ERK1/2 phosphorylation through AT2R, and inhibited the proinflammatory Ang II. Numerous inflammatory mediator factors, such as IL-1β, TNF-α, IL-6, TGF-β, and CXCL9, are produced and ultimately contribute to the cytokine storm and ALI [86,87]. Chen et al. [88] found that the spike protein of SARS-CoV-2 mediated viral entry through the binding to ACE2 on the cell surface. Menezes et al. [89] and Li et al. [90] hypothesized that immune cells, including monocytes, neutrophils, lymphocytes, and natural killer cells, could travel to infected areas after breaking endothelial and epithelial barriers. These immune cells could eliminate infected cells as well as alveolar exudates, leading to uncontrolled inflammation [89,90]. Patel et al. [91] found that patients who died from SARS-CoV-2 manifested massive infiltration of various immune cells in their alveolus, which formed a cytokine storm and resulted in lung failure (Figure 2).

## 4. ACE2, SARS-CoV-2, and Innate Immune Pathways

**Figure 3:**
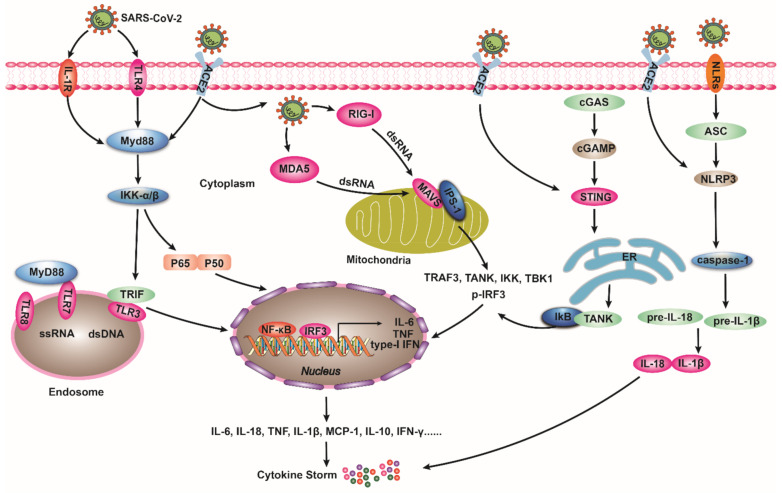
The major signal pathways of immunity against SARS-CoV2 infection. (i). TLRs primarily activate the transcription of NF-κB through MyD88 and TRIF-dependent pathways, which leads to the secretion of inflammatory factors and type-I IFN. (ii). RIG-I and MDA5 recognize the dsRNA in the cytoplasm and activate the downstream mitochondrial antiviral-signaling protein (MAVS) and IPS-1. Immune response is induced, including the activation of NF-κB and IRF3 transcription. (iii). cGAS identifies the dsDNA of the virus, thus inducing the production of the second messenger cyclic guanosine monophosphate adenosine monophosphate (cGAMP), which binds to STING on the endoplasmic reticulum membrane to cause an inflammatory response. As a member of the NLRs family, NLRP3 activates caspase-1, resulting in the cleavage of pre-IL-1β and pre-IL-18 to generate IL-1β and IL-18 in the lung inflammatory response. Abbreviations: TLRs, Toll-like receptors; MDA5, melanoma differentiation-associated protein5.

The association between the innate immune system and SARS-CoV-2 is crucial for regulating viral infection, disease progression, and prognosis [92,93]. Innate immunity relies on pattern recognition receptors (PRRs) by multiple cell surfaces or intracellular receptors and responds by signal transduction to effector molecules [94,95]. SARS-CoV-2 infection activates pattern recognition receptors on pulmonary epithelial cells, endothelial cells, macrophages, DCs, and other immune cells to produce cytokines [96,97]. Recognition receptors including Toll-like receptors (TLRs), retinoic acid-inducible gene I (RIG-I, melanoma differentiation-associated gene 5 (MDA5), cyclic guanosine phosphate adenosine phosphate synthase (cGAS), stimulator of interferon genes (STING), and nucleotide-binding oligomerization domain-like receptors (NLRs) play a significant role in antiviral defense against coronaviruses (Figure 3) [98,99,100].

### 4.1. TLRs Pathway

TLRs are a family of transmembrane receptors that are important in the immune system [101]. TLRs recognize SARS-CoV-2 and induce activation of the immune system and inflammation [98,102,103]. Elenad et al. [104] found that the ACE2/Ang-(1-7)/AT2R axis interact with TLRs to regulate the T lymphocyte response, thereby playing an anti-inflammatory role. TLRs have several family members, among which, TLR3, TLR7, and TLR8 are expressed on the surface of cellular endosomes, recognizing viruses as transmembrane receptors [105,106]. Xia et al. [107] found that Chinese herbal medicines show good affinity for SARS-CoV2 3CLpro and ACE2 and could inhibit tumor necrosis factor (TNF) and TLRs. Toll-like receptor 2 (TLR2) is a major pattern recognition receptor (PRR) and mediates viral infection via the TLR2/MyD88 pathway [108,109]. Dosch et al. [110] found that the S protein of SARS-CoV-2 could trigger activation and nucleus translocation of NF-κB and cause an inflammatory response in peripheral blood monocytes (PBMCs) through TLR2. Choudhury et al. [98] showed that S proteins of SARS-CoV-2 could also interact with hydrogen bonds and hydrophobic areas of TLR1, TLR4, and TLR6 besides ACE2. In addition, TLR4 initiated the key pathway to regulate ALI. Imai et al. [111] reported that mice infected with influenza virus hemagglutinin 5 neuraminidase 1 (H5N1), SARS, or *Bacillus anthracis* showed an excessive production of oxidized phospholipids (OxPAPC), which activated the TLR4–TRIIF–TRAF6 signaling pathway and released massive inflammatory factors from macrophages, inducing ALI. TLR4 knockdown impaired H5N1 infection in the lung and alleviated ALI [111]. TLR3 recognizes ligands to initiate the signaling through TIR-containing adaptor molecule (TICAM-1/TRIF), followed by the activation of NF-κB and interferon regulatory factor 3 (IRF3). These transcription factors modulate the release of type-I IFN from immune cells, mediating innate antiviral immunity [112,113]. Treatment with Poly I: C, a double-stranded RNA analog that interacts with TLR3, can directly activate alveolar macrophages and DCs, eliminating the inhibition of alveolar macrophages [114]. Moreover, Nakazono et al. [115] reported that Poly I:C could bind to TLR3 to activate IFN and the NF-κ B pathway, promoting SARS-CoV-2 entry into nasal mucosal epithelial cells to induce ACE2 expression. MyD88 (myeloid cell differentiation factor 88), a signal adaptor protein of TLR7 and TLR8, can recognize single-stranded ribonucleic acid (ssRNA) [116]. During SARS, MyD88 mediates the transmission of natural immune signals and recruits inflammatory cells to the lung and induces human plasma DCs (pDCs) to produce type-I IFN signaling through TLR7 to control rapid viral replication [117]. It was also found that SARS-CoV ssRNA had an intense immunological activity and could stimulate numerous IL-6, TNF-α, and IL-12 via TLR7 and TLR8 signaling pathways, which induced a cytokine storm in mice and finally caused ALI [118,119,120,121,122]. Studies have found that chemical compounds (PHA-408) could block TLRs and reduce inflammation [123]. In conclusion, blocking TLR7- and TLR8-mediated signaling pathways could inhibit the inflammatory response induced by a cytokine storm. However, whether this strategy could be applied for the treatment of coronavirus infection remains to be further studied.

### 4.2. RIG-I/MDA5 Pathway

RIG-I is a multidomain intracellular protein highly related to anti-RNA virus immunity [124,125]. The RIG-I/MDA5 signaling pathway mainly recognizes viral RNA in the cytoplasm and induces a natural immune response and inflammation, thereby controlling infection [126,127,128]. Studies have shown that SARS-CoV-2 RNA could trigger activation of the RIG-I-MAVS pathway and enhance the release of type-I IFN [129]. Yang et al. [130] found that after knocking out RIG-I, MDA5, or mitochondrial antiviral-signaling protein (MAVS), SARS-CoV-2-infected human endothelial cells produced notably less type-I/III IFN and the expression of ACE2 was increased. In alveolar epithelial cells, ORF9B inhibited the release of IFN, thereby inhibiting antiviral immunity [129]. Viral particles fuse with cellular membranes or vacuoles during their invasion to release dsRNA into the cytoplasm, and RIG-I/MDA5 can identify viral RNAs and catalyze their own activation [131]. Innate immunity is subsequently induced via downstream MAVS and IPS-1, activating multiple complexes, such as TRAF3, TANK, IKK, TBK1, and p-IRF3. This leads to the activation of NF-κB and IRF3, which participate in the transcription of type-I IFN and play antiviral functions in the initial stage of infection [112,132,133,134,135].

### 4.3. cGAS/STING Pathway

The cGAS/STING pathway stimulates type-I IFN during DNA and RNA viral infections, an essential factor in resisting viral infections [136,137]. DNA viruses, including adenovirus and vaccinia virus, can activate cGAS/STING, mice deficient in this pathway are more susceptive to viral infection [138]. During SARS-CoV-2 infection, cGAS binds with dsDNA to catalyze its transformation into cGAMP. cGAMP acts as a second messenger and it interacts with the adaptor protein STING on the endoplasmic reticulum (ER) membrane [139] to activate downstream kinase complex TANK-binding kinase 1 (TBK1) and inhibitor of κB protein kinase (IKK), resulting in nuclear translocation of IRF3 and NF-κB [140,141,142].

The cGAS/STING pathway protects host cells against RNA viruses. Fiachra et al. [143] found that in K18-hACE2-transfected mice with or without SARS-CoV-2 infection, intranasal administration of diazi-4 could activate STING, and inhibit viral replication in lung epithelial cells. Knockout of cGAS or STING genes in vitro or in vivo promoted large-scale replication of RNA viruses [144,145]. Applying STING agonist to influenza vaccine could improve its ability to resist multiple influenza viruses [146]. ORF3a of SARS-CoV-2 bound to STING and inhibited downstream signaling, restraining the nuclear accumulation of p65, preventing type-I IFN expression [137,147,148,149,150]. Berthelot et al. [151] found that the combination of SARS-CoV2 and ACE2 induced Ang II in mice, leading to overactivation of the STING pathway, which promoted tissue damage via monocyte-macrophage releasing interferon-β and tissue factors. cGAS-STING could not recognize viral RNA directly, but it could be activated by damaged host dsDNA, inducing the expression of type-I IFN and participating in the defense against SARS-CoV-2 infection with TLR3 and RIGI-MDA5 pathways [152,153,154].

### 4.4. NLRs Signaling Pathway

The NOD-like receptor (NLR) family is a family of immune receptors that identify pathogen-related cytoplasmic proteins. NOD-like receptor family pyrin domain containing 3 (NLRP3) is a component of the NLRs family, which plays a vital role in resisting viral infection in the lung by identifying viral RNA and subsequently activating the inflammasome [155]. Studies reported that SARS-CoV-2 activated NLRP3 after binding to ACE2 on vascular endothelial cells, inducing pyroptotic cell death, while the ACE2/Ang (1-7)/Mas axis regulated pyrolysis by inhibiting NLRP3 and eased the cytokine storm to exert a protective effect [156,157,158,159]. The NLRP3 inflammasome could be activated through the trans-Golgi network (TGN) owing to the stimulation of ion channels in the host cell membrane by viral porins. These viral porins, including the envelope (E) protein of the SARS-CoV2 virus and the M2 protein of the influenza virus, could subsequently activate caspase-1 [160,161,162]. NLRP3 activated by viral porins triggered pre-IL-1β and pre-IL-18 hydrolysis by caspase-1, generating excessive IL-1β, IL-18, and TNF-α and recruiting immune cells into the lung, leading to the death of infected cells. At the same time, reduced secretion of inflammatory factors was exhibited in NLRP3 knockout mice, which contributed to a higher mobility rate [155,160,163]. Studies have confirmed that patients infected with SARS and MERS had elevated levels of IL-1β and IL-18 in the lungs and lymphatic tissues, suggesting inflammasome activation [164,165]. Furthermore, the expression of TNF-α and IL-1β upon inflammasome complex activation promoted the accumulation of IL-6, which could act as one of the main impetuses of SARS-CoV-induced pulmonary inflammation and ARDS [160,166].

## 5. The Potential Therapeutic Role of ACE2 in COVID-19

Currently, there is no specific drug or vaccine for COVID-19. According to statistics, the mortality rate of severe COVID-19 patients is as high as 22–44% [72,167]. There is an urgent need for more effective treatments in the clinic. ACE2, as a binding protein of SARS-CoV2, not only plays a vital role in viral infection [168,169], but its dysfunction also leads to the weakness of the inflammatory inhibitory effect of ACE2/Ang-(1-7)/MASR axis, resulting in the aggravation of lung injury [170]. In addition, ACE2 is also a receptor protein for SARS-CoV-2-infected cells. Considering the potential treatment of ACE2-mediated COVID-19, some feasible treatments will be very important [171,172]. Therefore, ACE2 may play a certain therapeutic role on COVID-19 (Table 1).

### 5.1. Chloroquine

Studies have shown that chloroquine reduced viral infection by obstructing the binding of SARS-CoV-2 to ACE2 [173,174,175]. Gies et al. [172] found that chloroquine could destroy the terminal glycosylation of ACE2. This contributed to the conformational change of ACE2, therefore disturbing ACE2-SARS-CoV-2 binding and inhibiting viral replication in the host cells. Chloroquine also has anti-inflammatory and antiautophagy functions, which alleviate H5N1-induced lung injury in mice [176]. Therefore, chloroquine might have therapeutic effects on SARS. However, some studies have shown that chloroquine had no therapeutic effect on patients [177,178,179], and even had serious side effects and increased risk of death [180,181,182]. For these reasons, some hospitals have stopped using the drug.

### 5.2. ACEI or ARBs

Extensive attention has been attracted to whether ACEI/ARBs can be applied to the treatment of COVID-19 [183]. Neyrinck et al. [184] demonstrated that ACEI had a therapeutic effect on endotoxin-induced lung injury in rats. Melissa et al. [185] showed that ACEI and ARBs might improve ARDS by inhibiting the ACE/Ang II/AT1R signaling pathway. ARBs inhibited the canonical RAS pathway through angiotensin /Ang II/ATR1 axis; increasing ACE2, TLR-2, and IL-1β; and causing accumulation of reactive oxygen species (ROS), while ACE2 upregulation activates Ang-(1-7)/Mas pathway and inhibits inflammatory signals [186]. Studies identified that ARB could rescue SARS-CoV-2 spike or influenza virus-mediated ALI [187,188]. However, other studies inferred that there could be adverse aspects of using ACEI/ARB [189]. A recent study found that ACE2 was significantly upregulated after SARS-CoV and MERS-CoV infection, which could enhance their infection and transmission ability, and boosted the severity of COVID-19 [190]. However, ACEIs/ARBs were not associated with enhanced SARS-CoV-2 infection but led to decreased mortality [189,191]. Therefore, further investigations are still needed to demonstrate the function of ACEI/ARBs in COVID-19.

### 5.3. Recombinant ACE2

Recombinant ACE2, a recombinant soluble receptor that has a potential therapeutic effect on ALI, as it blocks the binding of SARS-CoV-2 to ACE2-expressing cells, thus avoiding viral infection [40,192,193,194,195]. Hoepel et al. [196] constructed recombinant proteins using an Fc fragment of human IgG1 together with the extracellular domain of either normal or mutant ACE2. Both recombinant proteins could interact with SARS-CoV-2 spike protein and therefore obstruct viral invasion. Monteil et al. [197] reported that human recombinant ACE2 (rhACE2) significantly prevented SARS-CoV-2 invasion, indicating that rhACE2 plays a role in the initial stage of the disease. However, whether this kind of drug is effective only for early infection remains uncertain [193]. Huang et al. [198] suggested that recombinant ACE2-Fc proteins not only worked as antibodies to block viral invasion and generate long-term immunity, but they could also act as a complement to decrease pulmonary ACE2 during infection, which improved the pathologic conditions of ARDS directly.

### 5.4. Vitamin D

Vitamin D has anti-inflammatory properties and plays a crucial role in immunity [199,200,201]. Li et al. [202,203] presented that 1,25(OH)_2_D3, the active form of vitamin D, also called calcitriol, was a negative regulator of RAS and inhibited renin synthesis. Xu et al. [204] confirmed that vitamin D could block ACE and Ang II expression and the increase of the ACE2 level in LPS-induced ALI. Mendonca et al. [205] found that activation of Nrf2 by vitamin D could reduce oxidative stress and inflammation, enhance innate immunity, and downregulate ACE2 to reduce the severity of SARS-CoV-2 infection.

**Table 1:** The potential therapeutic role of ACE2 in SARS-CoV-2-induced acute lung injury.

Drug	Major Outcome(s) Relates to ACE2 and SARS-CoV-2	References
Chloroquine	Chloroquine inhibited the binding of SARS-CoV-2 to ACE2, reducing the infection of the host cell by the virus. However, an increase in overall mortality was found in patients treated with chloroquine.	Ortiz MEetal., 2020 [173] Devaux et al., 2020 [174] Joseph et al., 2020 [182]
ACEI or ARBs	ACEI or ARBs inhibited the ACE/Ang/AT1R pathway to reduce inflammatory response and alleviate ARDS.	Neyrinck et al., 2009 [184] Melissa et al., 2021 [185] Yisireyili M et al., 2018 [186]
rhACE2	rhACE2 could bind to the spike protein by competing with ACE2 on the cell membrane surface, which on the one hand, inhibited the virus from infecting cells, on the other hand, rhACE2 could activate ACE2-Ang (1-7)-MasR pathway, reducing lung inflammation, and alleviating lung injury or ARDS.	Gheblawi et al., 2020 [40] Guzik et al., 2020 [192] Hoepel et al., 2021 [196]
Vitamin D	Vitamin D reduced oxidative stress and inflammation, enhancing innate immunity, and downregulating the expression of ACE2 to reduce the severity of SARS-CoV-2 infection.	Mendonca et al., 2020 [205]

## 6. Conclusions Remarks and Future Perspectives

In summary, RAS and ACE2/Ang- (1-7)/MasR axes play a critical role in ALI induced by SARS-CoV-2. Normal cellular immune responses have important protection against SARS-CoV-2 in lung host cells, but overexcited immune responses could induce cytokine storms, leading to cell death. This will eventually cause damage to the lung tissues and affect the normal respiratory function of the lung. Although SARS-CoV-2 and SARS-CoV are highly homologous, there are different characteristics between SARS-CoV-2, MERS-CoV, SARS-CoV, Ebola virus, and influenza A virus H1N1 infection. SARS-CoV-2 and SARS-CoV bind to ACE2 on the cell membrane using their spike protein. This mediates viral entry into cells, and on the other hand, may also destroy ACE2, resulting in an imbalance of RAS and ACE2/Ang- (1-7)/MasR. This dysregulation can exacerbate the disease and lead to ALI. Overreactive immune cells can generate cytokine storms, deteriorating multiple organs functions throughout the body. Therefore, blocking the spike binding to ACE2 or restoring the balance of RAS and ACE2/Ang-(1-7)/MasR are potential targets for developing specific drugs, antibodies, and vaccines for COVID-19 treatment. More studies on SARS-CoV and ACE2 are needed to understand the pathological mechanism of SARS-SoV-2 and to develop new preventive and treatment tools for COVID-19 in an effort to overcome the pandemic.

## Data Availability

Not applicable.

## Abbreviations

ACE2	Angiotensin-converting enzyme 2
ARBs	Angiotensin receptor blockers
ALI	Acute lung injury
AT1R	Angiotensin type 1 receptor
AT2R	Angiotensin 2 receptor
ACEI	Angiotensin-converting enzyme inhibitor
Ang II	Angiotensin II
COVID-19	Coronavirus disease
cGAS	Cyclic guanosine phosphate adenosine phosphate synthase
cGAMP	Cyclic guanosine monophosphate adenosine monophosphate
DCs	Dendritic cells
IRF3	Interferon regulatory factor 3
MDA5	Melanoma differentiation associated gene 5
MAVS	Mitochondrial antiviral-signaling protein
MasR	Mas receptor
MyD88	Myeloid cell differentiation factor 88
NLRs	Nucleotide-binding oligomerization domain-like receptors
NLRP3	NOD-like receptor family pyrin domain containing 3
PRRs	Pattern recognition receptors
RAS	Renin-angiotensin system
rhACE2	Recombinant human angiotensin converting enzyme 2
RIG-I	Retinoic acid-inducible gene I
SARS-CoV-2	Severe Acute Respiratory Syndrome Coronavirus 2
STING	Stimulator of interferon genes
TLRs	Toll-like receptors

## References

[1] Xu M., Wang D., Wang H., Zhang X., Liang T., Dai J., Li M., Zhang J., Zhang K., Xu D. (2020). COVID-19 diagnostic testing: Technology perspective. Clin. Transl. Med..

[2] Zhang Z., Wang X., Wei X., Zheng S.W., Lenhart B.J., Xu P., Li J., Pan J., Albrecht H., Liu C. (2021). Multiplex quantitative detection of SARS-CoV-2 specific IgG and IgM antibodies based on DNA-assisted nanopore sensing. Biosens. Bioelectron..

[3] Kola L., Kohrt B.A., Hanlon C., Naslund J.A., Sikander S., Balaji M., Benjet C., Cheung E.Y.L., Eaton J., Gonsalves P. (2021). COVID-19 mental health impact and responses in low-income and middle-income countries: Reimagining global mental health. Lancet Psychiatry.

[4] Huang C., Wang Y., Li X., Ren L., Zhao J., Hu Y., Zhang L., Fan G., Xu J., Gu X. (2020). Clinical features of patients infected with 2019 novel coronavirus in Wuhan, China. Lancet.

[5] Zhu N., Zhang D., Wang W., Li X., Yang B., Song J., Zhao X., Huang B., Shi W., Lu R. (2020). A Novel Coronavirus from Patients with Pneumonia in China, 2019. N. Engl. J. Med..

[6] Salter A., Fox R.J., Newsome S.D., Halper J., Li D.K.B., Kanellis P., Costello K., Bebo B., Rammohan K., Cutter G.R. (2021). Outcomes and Risk Factors Associated with SARS-CoV-2 Infection in a North American Registry of Patients with Multiple Sclerosis. JAMA Neurol..

[7] Ji L., Cao C., Gao Y., Zhang W., Xie Y., Duan Y., Kong S., You M., Ma R., Jiang L. (2020). Prognostic value of bedside lung ultrasound score in patients with COVID-19. Crit. Care.

[8] Chan S.W. (2020). Current and Future Direct-Acting Antivirals against COVID-19. Front. Microbiol..

[9] Xiao N., Nie M., Pang H., Wang B., Hu J., Meng X., Li K., Ran X., Long Q., Deng H. (2021). Integrated cytokine and metabolite analysis reveals immunometabolic reprogramming in COVID-19 patients with therapeutic implications. Nat. Commun..

[10] Rodda L.B., Netland J., Shehata L., Pruner K.B., Morawski P.A., Thouvenel C.D., Takehara K.K., Eggenberger J., Hemann E.A., Waterman H.R. (2021). Functional SARS-CoV-2-Specific Immune Memory Persists after Mild COVID-19. Cell.

[11] Livanos A.E., Jha D., Cossarini F., Gonzalez-Reiche A.S., Tokuyama M., Aydillo T., Parigi T.L., Ladinsky M.S., Ramos I., Dunleavy K. (2021). Intestinal Host Response to SARS-CoV-2 Infection and COVID-19 Outcomes in Patients with Gastrointestinal Symptoms. Gastroenterology.

[12] Vella L.A., Giles J.R., Baxter A.E., Oldridge D.A., Diorio C., Kuri-Cervantes L., Alanio C., Pampena M.B., Wu J.E., Chen Z. (2021). Deep immune profiling of MIS-C demonstrates marked but transient immune activation compared to adult and pediatric COVID-19. Sci. Immunol..

[13] Kai H., Kai M. (2020). Interactions of coronaviruses with ACE2, angiotensin II, and RAS inhibitors-lessons from available evidence and insights into COVID-19. Hypertens. Res..

[14] Pabalan N., Tharabenjasin P., Suntornsaratoon P., Jarjanazi H., Muanprasat C. (2021). Ethnic and age-specific acute lung injury/acute respiratory distress syndrome risk associated with angiotensin-converting enzyme insertion/deletion polymorphisms, implications for COVID-19: A meta-analysis. Infect. Genet. Evol..

[15] Cruces P., Diaz F., Puga A., Erranz B., Donoso A., Carvajal C., Wilhelm J., Repetto G.M. (2012). Angiotensin-converting enzyme insertion/deletion polymorphism is associated with severe hypoxemia in pediatric ARDS. Intensive Care Med..

[16] Montalvan V., Lee J., Bueso T., De Toledo J., Rivas K. (2020). Neurological manifestations of COVID-19 and other coronavirus infections: A systematic review. Clin. Neurol. Neurosurg..

[17] Datta P.K., Liu F., Fischer T., Rappaport J., Qin X. (2020). SARS-CoV-2 pandemic and research gaps: Understanding SARS-CoV-2 interaction with the ACE2 receptor and implications for therapy. Theranostics.

[18] Cozier G.E., Lubbe L., Sturrock E.D., Acharya K.R. (2021). Angiotensin-converting enzyme open for business: Structural insights into the subdomain dynamics. FEBS J..

[19] Davidson A.M., Wysocki J., Batlle D. (2020). Interaction of SARS-CoV-2 and Other Coronavirus with ACE (Angiotensin-Converting Enzyme)-2 as Their Main Receptor: Therapeutic Implications. Hypertension.

[20] Riordan J.F. (2003). Angiotensin-I-converting enzyme and its relatives. Genome Biol..

[21] Lieb W., Graf J., Gotz A., Konig I.R., Mayer B., Fischer M., Stritzke J., Hengstenberg C., Holmer S.R., Doring A. (2006). Association of angiotensin-converting enzyme 2 (ACE2) gene polymorphisms with parameters of left ventricular hypertrophy in men. Results of the MONICA Augsburg echocardiographic substudy. J. Mol. Med..

[22] Donoghue M., Hsieh F., Baronas E., Godbout K., Gosselin M., Stagliano N., Donovan M., Woolf B., Robison K., Jeyaseelan R. (2000). A novel angiotensin-converting enzyme-related carboxypeptidase (ACE2) converts angiotensin I to angiotensin 1-9. Circ. Res..

[23] Imai Y., Kuba K., Rao S., Huan Y., Guo F., Guan B., Yang P., Sarao R., Wada T., Leong-Poi H. (2005). Angiotensin-converting enzyme 2 protects from severe acute lung failure. Nature.

[24] Ferrario C.M., Chappell M.C. (2004). Novel angiotensin peptides. Cell Mol. Life Sci..

[25] Li X.C., Zhu D., Zheng X., Zhang J., Zhuo J.L. (2018). Intratubular and intracellular renin-angiotensin system in the kidney: A unifying perspective in blood pressure control. Clin. Sci..

[26] Prieto I., Villarejo A.B., Segarra A.B., Banegas I., Wangensteen R., Martinez-Canamero M., de Gasparo M., Vives F., Ramirez-Sanchez M. (2014). Brain, heart and kidney correlate for the control of blood pressure and water balance: Role of angiotensinases. Neuroendocrinology.

[27] Steckelings U.M., Sumners C. (2020). Correcting the imbalanced protective RAS in COVID-19 with angiotensin AT2-receptor agonists. Clin. Sci..

[28] Pedrosa M.A., Valenzuela R., Garrido-Gil P., Labandeira C.M., Navarro G., Franco R., Labandeira-Garcia J.L., Rodriguez-Perez A.I. (2021). Experimental data using candesartan and captopril indicate no double-edged sword effect in COVID-19. Clin. Sci..

[29] Ribeiro V.T., Cordeiro T.M.E., Filha R.D.S., Perez L.G., Caramelli P., Teixeira A.L., de Souza L.C., Simoes E.S.A.C. (2021). Circulating Angiotensin-(1-7) Is Reduced in Alzheimer’s Disease Patients and Correlates with White Matter Abnormalities: Results From a Pilot Study. Front. Neurosci..

[30] Hrenak J., Simko F. (2020). Renin-Angiotensin System: An Important Player in the Pathogenesis of Acute Respiratory Distress Syndrome. Int. J. Mol. Sci..

[31] Gnanenthiran S.R., Borghi C., Burger D., Charchar F., Poulter N.R., Schlaich M.P., Steckelings U.M., Stergiou G., Tomaszewski M., Unger T. (2021). Prospective meta-analysis protocol on randomised trials of renin-angiotensin system inhibitors in patients with COVID-19: An initiative of the International Society of Hypertension. BMJ Open.

[32] Passos-Silva D.G., Verano-Braga T., Santos R.A. (2013). Angiotensin-(1-7): Beyond the cardio-renal actions. Clin. Sci..

[33] Verma A., Shan Z., Lei B., Yuan L., Liu X., Nakagawa T., Grant M.B., Lewin A.S., Hauswirth W.W., Raizada M.K. (2012). ACE2 and Ang-(1-7) confer protection against development of diabetic retinopathy. Mol. Ther..

[34] Trougakos I.P., Stamatelopoulos K., Terpos E., Tsitsilonis O.E., Aivalioti E., Paraskevis D., Kastritis E., Pavlakis G.N., Dimopoulos M.A. (2021). Insights to SARS-CoV-2 life cycle, pathophysiology, and rationalized treatments that target COVID-19 clinical complications. J. Biomed. Sci..

[35] Chen I.C., Lin J.Y., Liu Y.C., Chai C.Y., Yeh J.L., Hsu J.H., Wu B.N., Dai Z.K. (2021). Angiotensin-Converting Enzyme 2 Activator Ameliorates Severe Pulmonary Hypertension in a Rat Model of Left Pneumonectomy Combined with VEGF Inhibition. Front. Med..

[36] Shim K.Y., Eom Y.W., Kim M.Y., Kang S.H., Baik S.K. (2018). Role of the renin-angiotensin system in hepatic fibrosis and portal hypertension. Korean J. Intern. Med..

[37] Fliser D., Buchholz K., Haller H., EUropean Trial on Olmesartan and Pravastatin in Inflammation and Atherosclerosis (EUTOPIA) Investigators (2004). Antiinflammatory effects of angiotensin II subtype 1 receptor blockade in hypertensive patients with microinflammation. Circulation.

[38] Khanna P., Soh H.J., Chen C.H., Saxena R., Amin S., Naughton M., Joslin P.N., Moore A., Bakouny Z., O’Callaghan C. (2021). ACE2 abrogates tumor resistance to VEGFR inhibitors suggesting angiotensin-(1-7) as a therapy for clear cell renal cell carcinoma. Sci. Transl. Med..

[39] Keidar S., Kaplan M., Gamliel-Lazarovich A. (2007). ACE2 of the heart: From angiotensin I to angiotensin (1-7). Cardiovasc. Res..

[40] Gheblawi M., Wang K., Viveiros A., Nguyen Q., Zhong J.C., Turner A.J., Raizada M.K., Grant M.B., Oudit G.Y. (2020). Angiotensin-Converting Enzyme 2: SARS-CoV-2 Receptor and Regulator of the Renin-Angiotensin System: Celebrating the 20th Anniversary of the Discovery of ACE2. Circ. Res..

[41] Knight A.C., Montgomery S.A., Fletcher C.A., Baxter V.K. (2021). Mouse Models for the Study of SARS-CoV-2 Infection. Comp. Med..

[42] Zhou X., Ma F., Xie J., Yuan M., Li Y., Shaabani N., Zhao F., Huang D., Wu N.C., Lee C.D. (2021). Diverse immunoglobulin gene usage and convergent epitope targeting in neutralizing antibody responses to SARS-CoV-2. Cell Rep..

[43] South A.M., Shaltout H.A., Washburn L.K., Hendricks A.S., Diz D.I., Chappell M.C. (2019). Fetal programming and the angiotensin-(1-7) axis: A review of the experimental and clinical data. Clin. Sci..

[44] Kuba K., Imai Y., Rao S., Gao H., Guo F., Guan B., Huan Y., Yang P., Zhang Y., Deng W. (2005). A crucial role of angiotensin converting enzyme 2 (ACE2) in SARS coronavirus-induced lung injury. Nat. Med..

[45] Kuba K., Imai Y., Rao S., Jiang C., Penninger J.M. (2006). Lessons from SARS: Control of acute lung failure by the SARS receptor ACE2. J. Mol. Med..

[46] Wosten-van Asperen R.M., Lutter R., Specht P.A., Moll G.N., van Woensel J.B., van der Loos C.M., van Goor H., Kamilic J., Florquin S., Bos A.P. (2011). Acute respiratory distress syndrome leads to reduced ratio of ACE/ACE2 activities and is prevented by angiotensin-(1-7) or an angiotensin II receptor antagonist. J. Pathol..

[47] Huentelman M.J., Zubcevic J., Hernandez Prada J.A., Xiao X., Dimitrov D.S., Raizada M.K., Ostrov D.A. (2004). Structure-based discovery of a novel angiotensin-converting enzyme 2 inhibitor. Hypertension.

[48] Khan A., Benthin C., Zeno B., Albertson T.E., Boyd J., Christie J.D., Hall R., Poirier G., Ronco J.J., Tidswell M. (2017). A pilot clinical trial of recombinant human angiotensin-converting enzyme 2 in acute respiratory distress syndrome. Crit. Care.

[49] Chen Y., Qu L., Li Y., Chen C., He W., Shen L., Zhang R. (2021). Glycyrrhizic Acid Alleviates Lipopolysaccharide (LPS)-Induced Acute Lung Injury by Regulating Angiotensin-Converting Enzyme-2 (ACE2) and Caveolin-1 Signaling Pathway. Inflammation.

[50] Huang K., Zhang P., Zhang Z., Youn J.Y., Wang C., Zhang H., Cai H. (2021). Traditional Chinese Medicine (TCM) in the treatment of COVID-19 and other viral infections: Efficacies and mechanisms. Pharmacol. Ther..

[51] Meydan C., Madrer N., Soreq H. (2020). The Neat Dance of COVID-19: NEAT1, DANCR, and Co-Modulated Cholinergic RNAs Link to Inflammation. Front. Immunol..

[52] Gao Y.L., Du Y., Zhang C., Cheng C., Yang H.Y., Jin Y.F., Duan G.C., Chen S.Y. (2020). Role of Renin-Angiotensin System in Acute Lung Injury Caused by Viral Infection. Infect. Drug Resist..

[53] Gerard L., Lecocq M., Bouzin C., Hoton D., Schmit G., Pereira J.P., Montiel V., Plante-Bordeneuve T., Laterre P.F., Pilette C. (2021). Increased Angiotensin-Converting Enzyme 2 and Loss of Alveolar Type II Cells in COVID-19 Related ARDS. Am. J. Respir. Crit. Care Med..

[54] Asaka M.N., Utsumi D., Kamada H., Nagata S., Nakachi Y., Yamaguchi T., Kawaoka Y., Kuba K., Yasutomi Y. (2021). Highly susceptible SARS-CoV-2 model in CAG promoter-driven hACE2-transgenic mice. JCI Insight.

[55] Herman-Edelstein M., Guetta T., Barnea A., Waldman M., Ben-Dor N., Barak Y., Kornowski R., Arad M., Hochhauser E., Aravot D. (2021). Expression of the SARS-CoV-2 receptorACE2 in human heart is associated with uncontrolled diabetes, obesity, and activation of the renin angiotensin system. Cardiovasc. Diabetol..

[56] Savoia C., Volpe M., Kreutz R. (2021). Hypertension, a Moving Target in COVID-19: Current Views and Perspectives. Circ. Res..

[57] Rico-Mesa J.S., White A., Anderson A.S. (2020). Outcomes in Patients with COVID-19 Infection Taking ACEI/ARB. Curr. Cardiol. Rep..

[58] Chu C., Zeng S., Hasan A.A., Hocher C.F., Kramer B.K., Hocher B. (2020). Comparison of infection risks and clinical outcomes in patients with and without SARS-CoV-2 lung infection under renin-angiotensin-aldosterone system blockade: Systematic review and meta-analysis. Br. J. Clin. Pharmacol..

[59] Lee H.W., Yoon C.H., Jang E.J., Lee C.H. (2021). Renin-angiotensin system blocker and outcomes of COVID-19: A systematic review and meta-analysis. Thorax.

[60] Xu J., Teng Y., Shang L., Gu X., Fan G., Chen Y., Tian R., Zhang S., Cao B. (2020). The Effect of Prior ACEI/ARB Treatment on COVID-19 Susceptibility and Outcome: A Systematic Review and Meta-Analysis. Clin. Infect. Dis..

[61] Volpe M., Patrono C. (2021). A randomized trial supports the recommendation to continue treatment with ACEi or ARBs during hospitalization for COVID-19. Eur. Heart J..

[62] Nieto-Torres J.L., Verdia-Baguena C., Jimenez-Guardeno J.M., Regla-Nava J.A., Castano-Rodriguez C., Fernandez-Delgado R., Torres J., Aguilella V.M., Enjuanes L. (2015). Severe acute respiratory syndrome coronavirus E protein transports calcium ions and activates the NLRP3 inflammasome. Virology.

[63] Jimenez-Guardeno J.M., Nieto-Torres J.L., DeDiego M.L., Regla-Nava J.A., Fernandez-Delgado R., Castano-Rodriguez C., Enjuanes L. (2014). The PDZ-binding motif of severe acute respiratory syndrome coronavirus envelope protein is a determinant of viral pathogenesis. PLoS Pathog.

[64] Teoh K.T., Siu Y.L., Chan W.L., Schluter M.A., Liu C.J., Peiris J.S., Bruzzone R., Margolis B., Nal B. (2010). The SARS coronavirus E protein interacts with PALS1 and alters tight junction formation and epithelial morphogenesis. Mol. Biol. Cell.

[65] Muus C., Luecken M.D., Eraslan G., Sikkema L., Waghray A., Heimberg G., Kobayashi Y., Vaishnav E.D., Subramanian A., Smillie C. (2021). Single-cell meta-analysis of SARS-CoV-2 entry genes across tissues and demographics. Nat. Med..

[66] Wang S., Qiu Z., Hou Y., Deng X., Xu W., Zheng T., Wu P., Xie S., Bian W., Zhang C. (2021). AXL is a candidate receptor for SARS-CoV-2 that promotes infection of pulmonary and bronchial epithelial cells. Cell Res..

[67] McCracken I.R., Saginc G., He L., Huseynov A., Daniels A., Fletcher S., Peghaire C., Kalna V., Andaloussi-Mae M., Muhl L. (2021). Lack of Evidence of Angiotensin-Converting Enzyme 2 Expression and Replicative Infection by SARS-CoV-2 in Human Endothelial Cells. Circulation.

[68] Ramassamy J.L., Tortevoye P., Ntab B., Seve B., Carles G., Gaquiere D., Madec Y., Fontanet A., Gessain A. (2020). Adult T-cell leukemia/lymphoma incidence rate in French Guiana: A prospective cohort of women infected with HTLV-1. Blood Adv..

[69] Ma D., Liu S., Hu L., He Q., Shi W., Yan D., Cao Y., Zhang G., Wang Z., Wu J. (2021). Single-cell RNA sequencing identify SDCBP in ACE2-positive bronchial epithelial cells negatively correlates with COVID-19 severity. J. Cell Mol. Med..

[70] Dikshith T.S., Raizada R.B., Kumar S.N., Srivastava M.K., Kaushal R.A., Singh R.P., Gupta K.P. (1988). Effect of repeated dermal application of endosulfan to rats. Vet. Hum. Toxicol..

[71] Aboudounya M.M., Heads R.J. (2021). COVID-19 and Toll-Like Receptor 4 (TLR4): SARS-CoV-2 May Bind and Activate TLR4 to Increase ACE2 Expression, Facilitating Entry and Causing Hyperinflammation. Mediat. Inflamm..

[72] Yang X., Yu Y., Xu J., Shu H., Xia J., Liu H., Wu Y., Zhang L., Yu Z., Fang M. (2020). Clinical course and outcomes of critically ill patients with SARS-CoV-2 pneumonia in Wuhan, China: A single-centered, retrospective, observational study. Lancet Respir. Med..

[73] Obukhov A.G., Stevens B.R., Prasad R., Li Calzi S., Boulton M.E., Raizada M.K., Oudit G.Y., Grant M.B. (2020). SARS-CoV-2 Infections and ACE2: Clinical Outcomes Linked with Increased Morbidity and Mortality in Individuals with Diabetes. Diabetes.

[74] Zhang X., Zheng J., Yan Y., Ruan Z., Su Y., Wang J., Huang H., Zhang Y., Wang W., Gao J. (2019). Angiotensin-converting enzyme 2 regulates autophagy in acute lung injury through AMPK/mTOR signaling. Arch. Biochem. Biophys..

[75] Caci G., Albini A., Malerba M., Noonan D.M., Pochetti P., Polosa R. (2020). COVID-19 and Obesity: Dangerous Liaisons. J. Clin. Med..

[76] De Oliveira A.P., Lopes A.L.F., Pacheco G., de Sa Guimaraes Noleto I.R., Nicolau L.A.D., Medeiros J.V.R. (2020). Premises among SARS-CoV-2, dysbiosis and diarrhea: Walking through the ACE2/mTOR/autophagy route. Med. Hypotheses.

[77] Li Y., Cao Y., Zeng Z., Liang M., Xue Y., Xi C., Zhou M., Jiang W. (2015). Angiotensin-converting enzyme 2/angiotensin-(1-7)/Mas axis prevents lipopolysaccharide-induced apoptosis of pulmonary microvascular endothelial cells by inhibiting JNK/NF-kappaB pathways. Sci. Rep..

[78] Uhal B.D., Li X., Xue A., Gao X., Abdul-Hafez A. (2011). Regulation of alveolar epithelial cell survival by the ACE-2/angiotensin 1-7/Mas axis. Am. J. Physiol. Lung Cell Mol. Physiol..

[79] Hasan H.F., Elgazzar E.M., Mostafa D.M. (2020). Diminazene aceturate extenuate the renal deleterious consequences of angiotensin-II induced by gamma-irradiation through boosting ACE2 signaling cascade. Life Sci..

[80] Wang J., Kaplan N., Wysocki J., Yang W., Lu K., Peng H., Batlle D., Lavker R.M. (2020). The ACE2-deficient mouse: A model for a cytokine storm-driven inflammation. FASEB J..

[81] Shen L., Mo H., Cai L., Kong T., Zheng W., Ye J., Qi J., Xiao Z. (2009). Losartan prevents sepsis-induced acute lung injury and decreases activation of nuclear factor kappaB and mitogen-activated protein kinases. Shock.

[82] Sharif-Askari N.S., Sharif-Askari F.S., Mdkhana B., Hussain Alsayed H.A., Alsafar H., Alrais Z.F., Hamid Q., Halwani R. (2021). Upregulation of oxidative stress gene markers during SARS-CoV-2 viral infection. Free Radic. Biol. Med..

[83] Chi Y., Ge Y., Wu B., Zhang W., Wu T., Wen T., Liu J., Guo X., Huang C., Jiao Y. (2020). Serum Cytokine and Chemokine Profile in Relation to the Severity of Coronavirus Disease 2019 in China. J. Infect. Dis..

[84] Zhang W., Dai H., Lin F., Zhao C., Wang X., Zhang S., Ge W., Pei S., Pan L. (2020). Ly-6C(high) inflammatory-monocyte recruitment is regulated by p38 MAPK/MCP-1 activation and promotes ventilator-induced lung injury. Int. Immunopharmacol..

[85] Robinson D.P., Hall O.J., Nilles T.L., Bream J.H., Klein S.L. (2014). 17beta-estradiol protects females against influenza by recruiting neutrophils and increasing virus-specific CD8 T cell responses in the lungs. J. Virol..

[86] Li S., Zhang Y., Guan Z., Li H., Ye M., Chen X., Shen J., Zhou Y., Shi Z.L., Zhou P. (2020). SARS-CoV-2 triggers inflammatory responses and cell death through caspase-8 activation. Signal Transduct. Target. Ther..

[87] Lee W., Ahn J.H., Park H.H., Kim H.N., Kim H., Yoo Y., Shin H., Hong K.S., Jang J.G., Park C.G. (2020). COVID-19-activated SREBP2 disturbs cholesterol biosynthesis and leads to cytokine storm. Signal Transduct. Target. Ther..

[88] Chen H., Kang Y., Duan M., Hou T. (2021). Regulation Mechanism for the Binding between the SARS-CoV-2 Spike Protein and Host Angiotensin-Converting Enzyme II. J. Phys. Chem. Lett..

[89] Menezes M.C.S., Pestana D.V.S., Gameiro G.R., da Silva L.F.F., Baron E., Rouby J.J., Auler J.O.C. (2021). SARS-CoV-2 pneumonia-receptor binding and lung immunopathology: A narrative review. Crit. Care.

[90] Li H., Liu L., Zhang D., Xu J., Dai H., Tang N., Su X., Cao B. (2020). SARS-CoV-2 and viral sepsis: Observations and hypotheses. Lancet.

[91] Patel M., Shahjin F., Cohen J.D., Hasan M., Machhi J., Chugh H., Singh S., Das S., Kulkarni T.A., Herskovitz J. (2021). The immunopathobiology of SARS-CoV-2 infection. FEMS Microbiol. Rev..

[92] Azkur A.K., Akdis M., Azkur D., Sokolowska M., van de Veen W., Bruggen M.C., O’Mahony L., Gao Y., Nadeau K., Akdis C.A. (2020). Immune response to SARS-CoV-2 and mechanisms of immunopathological changes in COVID-19. Allergy.

[93] Schijns V., Lavelle E.C. (2020). Prevention and treatment of COVID-19 disease by controlled modulation of innate immunity. Eur. J. Immunol..

[94] Chan J.F., To K.K., Tse H., Jin D.Y., Yuen K.Y. (2013). Interspecies transmission and emergence of novel viruses: Lessons from bats and birds. Trends Microbiol..

[95] Frieman M., Heise M., Baric R. (2008). SARS coronavirus and innate immunity. Virus Res..

[96] Jung S., Potapov I., Chillara S., Del Sol A. (2021). Leveraging systems biology for predicting modulators of inflammation in patients with COVID-19. Sci. Adv..

[97] Di Gioacchino A., Sulc P., Komarova A.V., Greenbaum B.D., Monasson R., Cocco S. (2021). The Heterogeneous Landscape and Early Evolution of Pathogen-Associated CpG Dinucleotides in SARS-CoV-2. Mol. Biol. Evol..

[98] Choudhury A., Mukherjee S. (2020). In silico studies on the comparative characterization of the interactions of SARS-CoV-2 spike glycoprotein with ACE-2 receptor homologs and human TLRs. J. Med. Virol..

[99] Li S., Kuang M., Chen L., Li Y., Liu S., Du H., Cao L., You F. (2021). The mitochondrial protein ERAL1 suppresses RNA virus infection by facilitating RIG-I-like receptor signaling. Cell Rep..

[100] Kienes I., Weidl T., Mirza N., Chamaillard M., Kufer T.A. (2021). Role of NLRs in the Regulation of Type I Interferon Signaling, Host Defense and Tolerance to Inflammation. Int. J. Mol. Sci..

[101] Fitzgerald K.A., Kagan J.C. (2020). Toll-like Receptors and the Control of Immunity. Cell.

[102] Zhao Y., Kuang M., Li J., Zhu L., Jia Z., Guo X., Hu Y., Kong J., Yin H., Wang X. (2021). SARS-CoV-2 spike protein interacts with and activates TLR41. Cell Res..

[103] Bhattacharya M., Sharma A.R., Mallick B., Sharma G., Lee S.S., Chakraborty C. (2020). Immunoinformatics approach to understand molecular interaction between multi-epitopic regions of SARS-CoV-2 spike-protein with TLR4/MD-2 complex. Infect. Genet. Evol..

[104] Cantero-Navarro E., Fernandez-Fernandez B., Ramos A.M., Rayego-Mateos S., Rodrigues-Diez R.R., Sanchez-Nino M.D., Sanz A.B., Ruiz-Ortega M., Ortiz A. (2021). Renin-angiotensin system and inflammation update. Mol. Cell Endocrinol..

[105] Zong Z., Zhang Z., Wu L., Zhang L., Zhou F. (2021). The Functional Deubiquitinating Enzymes in Control of Innate Antiviral Immunity. Adv. Sci..

[106] Li L., Acioglu C., Heary R.F., Elkabes S. (2021). Role of astroglial toll-like receptors (TLRs) in central nervous system infections, injury and neurodegenerative diseases. Brain Behav. Immun..

[107] Xia S., Zhong Z., Gao B., Vong C.T., Lin X., Cai J., Gao H., Chan G., Li C. (2021). The important herbal pair for the treatment of COVID-19 and its possible mechanisms. Chin. Med..

[108] Gianni T., Campadelli-Fiume G. (2014). The epithelial alphavbeta3-integrin boosts the MYD88-dependent TLR2 signaling in response to viral and bacterial components. PLoS Pathog..

[109] Qian Y., Lei T., Patel P., Lee C., Monaghan-Nichols P., Xin H.B., Qiu J., Fu M. (2021). Direct activation of endothelial cells by SARS-CoV-2 nucleocapsid protein is blocked by Simvastatin. bioRxiv.

[110] Dosch S.F., Mahajan S.D., Collins A.R. (2009). SARS coronavirus spike protein-induced innate immune response occurs via activation of the NF-kappaB pathway in human monocyte macrophages in vitro. Virus Res..

[111] Imai Y., Kuba K., Neely G.G., Yaghubian-Malhami R., Perkmann T., van Loo G., Ermolaeva M., Veldhuizen R., Leung Y.H., Wang H. (2008). Identification of oxidative stress and Toll-like receptor 4 signaling as a key pathway of acute lung injury. Cell.

[112] Mahla R.S., Reddy M.C., Prasad D.V., Kumar H. (2013). Sweeten PAMPs: Role of Sugar Complexed PAMPs in Innate Immunity and Vaccine Biology. Front. Immunol..

[113] Sallenave J.M., Guillot L. (2020). Innate Immune Signaling and Proteolytic Pathways in the Resolution or Exacerbation of SARS-CoV-2 in COVID-19: Key Therapeutic Targets?. Front. Immunol..

[114] Zhao J., Zhao J., Van Rooijen N., Perlman S. (2009). Evasion by stealth: Inefficient immune activation underlies poor T cell response and severe disease in SARS-CoV-infected mice. PLoS Pathog..

[115] Nakazono A., Nakamaru Y., Ramezanpour M., Kondo T., Watanabe M., Hatakeyama S., Kimura S., Honma A., Wormald P.J., Vreugde S. (2021). Fluticasone Propionate Suppresses Poly(I:C)-Induced ACE2 in Primary Human Nasal Epithelial Cells. Front. Cell Infect. Microbiol..

[116] Meas H.Z., Haug M., Beckwith M.S., Louet C., Ryan L., Hu Z., Landskron J., Nordbo S.A., Tasken K., Yin H. (2020). Sensing of HIV-1 by TLR8 activates human T cells and reverses latency. Nat. Commun..

[117] Barrat F.J., Su L. (2019). A pathogenic role of plasmacytoid dendritic cells in autoimmunity and chronic viral infection. J. Exp. Med..

[118] Li Y., Chen M., Cao H., Zhu Y., Zheng J., Zhou H. (2013). Extraordinary GU-rich single-strand RNA identified from SARS coronavirus contributes an excessive innate immune response. Microbes Infect..

[119] Lee I.H., Lee J.W., Kong S.W. (2020). A survey of genetic variants in SARS-CoV-2 interacting domains of ACE2, TMPRSS2 and TLR3/7/8 across populations. Infect. Genet. Evol..

[120] Angelopoulou A., Alexandris N., Konstantinou E., Mesiakaris K., Zanidis C., Farsalinos K., Poulas K. (2020). Imiquimod—A toll like receptor 7 agonist—Is an ideal option for management of COVID 19. Environ. Res..

[121] Moreno-Eutimio M.A., Lopez-Macias C., Pastelin-Palacios R. (2020). Bioinformatic analysis and identification of single-stranded RNA sequences recognized by TLR7/8 in the SARS-CoV-2, SARS-CoV, and MERS-CoV genomes. Microbes Infect..

[122] Sharma J., Parsai K., Raghuwanshi P., Ali S.A., Tiwari V., Bhargava A., Mishra P.K. (2021). Emerging role of mitochondria in airborne particulate matter-induced immunotoxicity. Environ. Pollut..

[123] Lai C.Y., Su Y.W., Lin K.I., Hsu L.C., Chuang T.H. (2017). Natural Modulators of Endosomal Toll-Like Receptor-Mediated Psoriatic Skin Inflammation. J. Immunol. Res..

[124] Myong S., Cui S., Cornish P.V., Kirchhofer A., Gack M.U., Jung J.U., Hopfner K.P., Ha T. (2009). Cytosolic viral sensor RIG-I is a 5′-triphosphate-dependent translocase on double-stranded RNA. Science.

[125] Chiang C., Liu G., Gack M.U. (2021). Viral Evasion of RIG-I-Like Receptor-Mediated Immunity through Dysregulation of Ubiquitination and ISGylation. Viruses.

[126] Errett J.S., Gale M. (2015). Emerging complexity and new roles for the RIG-I-like receptors in innate antiviral immunity. Virol. Sin..

[127] Loo Y.M., Gale M. (2011). Immune signaling by RIG-I-like receptors. Immunity.

[128] Esser-Nobis K., Hatfield L.D., Gale M. (2020). Spatiotemporal dynamics of innate immune signaling via RIG-I-like receptors. Proc. Natl. Acad. Sci. USA.

[129] Wu J., Shi Y., Pan X., Wu S., Hou R., Zhang Y., Zhong T., Tang H., Du W., Wang L. (2021). SARS-CoV-2 ORF9b inhibits RIG-I-MAVS antiviral signaling by interrupting K63-linked ubiquitination of NEMO. Cell Rep..

[130] Yang D., Geng T., Harrison A.G., Wang P. (2021). Differential roles of RIG-I-like receptors in SARS-CoV-2 infection. bioRxiv.

[131] Onomoto K., Onoguchi K., Yoneyama M. (2021). Regulation of RIG-I-like receptor-mediated signaling: Interaction between host and viral factors. Cell Mol. Immunol..

[132] Stuart J.D., Holm G.H., Boehme K.W. (2018). Differential Delivery of Genomic Double-Stranded RNA Causes Reovirus Strain-Specific Differences in Interferon Regulatory Factor 3 Activation. J. Virol..

[133] Lei Y., Moore C.B., Liesman R.M., O’Connor B.P., Bergstralh D.T., Chen Z.J., Pickles R.J., Ting J.P. (2009). MAVS-mediated apoptosis and its inhibition by viral proteins. PLoS ONE.

[134] Kash J.C., Muhlberger E., Carter V., Grosch M., Perwitasari O., Proll S.C., Thomas M.J., Weber F., Klenk H.D., Katze M.G. (2006). Global suppression of the host antiviral response by Ebola- and Marburgviruses: Increased antagonism of the type I interferon response is associated with enhanced virulence. J. Virol..

[135] Zheng Y., Zhuang M.W., Han L., Zhang J., Nan M.L., Zhan P., Kang D., Liu X., Gao C., Wang P.H. (2020). Severe acute respiratory syndrome coronavirus 2 (SARS-CoV-2) membrane (M) protein inhibits type I and III interferon production by targeting RIG-I/MDA-5 signaling. Signal Transduct. Target. Ther..

[136] Abe T., Marutani Y., Shoji I. (2019). Cytosolic DNA-sensing immune response and viral infection. Microbiol. Immunol..

[137] Maringer K., Fernandez-Sesma A. (2014). Message in a bottle: Lessons learned from antagonism of STING signalling during RNA virus infection. Cytokine Growth Factor Rev..

[138] Ma Z., Damania B. (2016). The cGAS-STING Defense Pathway and Its Counteraction by Viruses. Cell Host Microbe.

[139] Berthelot J.M., Liote F., Maugars Y., Sibilia J. (2020). Lymphocyte Changes in Severe COVID-19: Delayed Over-Activation of STING?. Front. Immunol..

[140] Barber G.N. (2015). STING: Infection, inflammation and cancer. Nat. Rev. Immunol..

[141] Li T., Chen Z.J. (2018). The cGAS-cGAMP-STING pathway connects DNA damage to inflammation, senescence, and cancer. J. Exp. Med..

[142] Hoong B.Y.D., Gan Y.H., Liu H., Chen E.S. (2020). cGAS-STING pathway in oncogenesis and cancer therapeutics. Oncotarget.

[143] Humphries F., Shmuel-Galia L., Jiang Z., Wilson R., Landis P., Ng S.L., Fitzgerald K.A. (2021). A diamidobenzimidazole STING agonist protects against SARS-CoV-2 infection. Sci. Immunol..

[144] Aguirre S., Luthra P., Sanchez-Aparicio M.T., Maestre A.M., Patel J., Lamothe F., Fredericks A.C., Tripathi S., Zhu T., Pintado-Silva J. (2017). Dengue virus NS2B protein targets cGAS for degradation and prevents mitochondrial DNA sensing during infection. Nat. Microbiol..

[145] Schoggins J.W., MacDuff D.A., Imanaka N., Gainey M.D., Shrestha B., Eitson J.L., Mar K.B., Richardson R.B., Ratushny A.V., Litvak V. (2014). Pan-viral specificity of IFN-induced genes reveals new roles for cGAS in innate immunity. Nature.

[146] Wang J., Li P., Yu Y., Fu Y., Jiang H., Lu M., Sun Z., Jiang S., Lu L., Wu M.X. (2020). Pulmonary surfactant-biomimetic nanoparticles potentiate heterosubtypic influenza immunity. Science.

[147] Rui Y., Su J., Shen S., Hu Y., Huang D., Zheng W., Lou M., Shi Y., Wang M., Chen S. (2021). Unique and complementary suppression of cGAS-STING and RNA sensing- triggered innate immune responses by SARS-CoV-2 proteins. Signal Transduct. Target. Ther..

[148] Sun L., Xing Y., Chen X., Zheng Y., Yang Y., Nichols D.B., Clementz M.A., Banach B.S., Li K., Baker S.C. (2012). Coronavirus papain-like proteases negatively regulate antiviral innate immune response through disruption of STING-mediated signaling. PLoS ONE.

[149] Clementz M.A., Chen Z., Banach B.S., Wang Y., Sun L., Ratia K., Baez-Santos Y.M., Wang J., Takayama J., Ghosh A.K. (2010). Deubiquitinating and interferon antagonism activities of coronavirus papain-like proteases. J. Virol..

[150] Ni G., Ma Z., Damania B. (2018). cGAS and STING: At the intersection of DNA and RNA virus-sensing networks. PLoS Pathog..

[151] Berthelot J.M., Drouet L., Liote F. (2020). Kawasaki-like diseases and thrombotic coagulopathy in COVID-19: Delayed over-activation of the STING pathway?. Emerg. Microbes Infect..

[152] Qiao Y., Zhu S., Deng S., Zou S.S., Gao B., Zang G., Wu J., Jiang Y., Liu Y.J., Chen J. (2020). Human Cancer Cells Sense Cytosolic Nucleic Acids Through the RIG-I-MAVS Pathway and cGAS-STING Pathway. Front. Cell Dev. Biol..

[153] Mdkhana B., Saheb Sharif-Askari N., Ramakrishnan R.K., Goel S., Hamid Q., Halwani R. (2021). Nucleic Acid-Sensing Pathways During SARS-CoV-2 Infection: Expectations versus Reality. J. Inflamm. Res..

[154] Levy R., Bastard P., Lanternier F., Lecuit M., Zhang S.Y., Casanova J.L. (2021). Correction to: IFN-alpha2a Therapy in Two Patients with Inborn Errors of TLR3 and IRF3 Infected with SARS-CoV-2. J. Clin. Immunol..

[155] Spel L., Martinon F. (2021). Detection of viruses by inflammasomes. Curr. Opin. Virol..

[156] Huang H., Wang J., Liu Z., Gao F. (2020). The angiotensin-converting enzyme 2/angiotensin (1-7)/mas axis protects against pyroptosis in LPS-induced lung injury by inhibiting NLRP3 activation. Arch. Biochem. Biophys..

[157] Ratajczak M.Z., Bujko K., Ciechanowicz A., Sielatycka K., Cymer M., Marlicz W., Kucia M. (2021). SARS-CoV-2 Entry Receptor ACE2 Is Expressed on Very Small CD45(-) Precursors of Hematopoietic and Endothelial Cells and in Response to Virus Spike Protein Activates the Nlrp3 Inflammasome. Stem Cell Rev. Rep..

[158] You Y., Huang Y., Wang D., Li Y., Wang G., Jin S., Zhu X., Wu B., Du X., Li X. (2019). Angiotensin (1–7) inhibits arecoline-induced migration and collagen synthesis in human oral myofibroblasts via inhibiting NLRP3 inflammasome activation. J. Cell Physiol..

[159] Ratajczak M.Z., Kucia M. (2020). SARS-CoV-2 infection and overactivation of Nlrp3 inflammasome as a trigger of cytokine “storm” and risk factor for damage of hematopoietic stem cells. Leukemia.

[160] Zhao C., Zhao W. (2020). NLRP3 Inflammasome-A Key Player in Antiviral Responses. Front. Immunol..

[161] Van Den Eeckhout B., Van Hoecke L., Burg E., Van Lint S., Peelman F., Kley N., Uze G., Saelens X., Tavernier J., Gerlo S. (2020). Specific targeting of IL-1beta activity to CD8(+) T cells allows for safe use as a vaccine adjuvant. NPJ Vaccines.

[162] Ichinohe T., Pang I.K., Iwasaki A. (2010). Influenza virus activates inflammasomes via its intracellular M2 ion channel. Nat. Immunol..

[163] Thomas P.G., Dash P., Aldridge J.R., Ellebedy A.H., Reynolds C., Funk A.J., Martin W.J., Lamkanfi M., Webby R.J., Boyd K.L. (2009). The intracellular sensor NLRP3 mediates key innate and healing responses to influenza A virus via the regulation of caspase-1. Immunity.

[164] Chen I.Y., Moriyama M., Chang M.F., Ichinohe T. (2019). Severe Acute Respiratory Syndrome Coronavirus Viroporin 3a Activates the NLRP3 Inflammasome. Front. Microbiol..

[165] Triantafilou K., Triantafilou M. (2014). Ion flux in the lung: Virus-induced inflammasome activation. Trends Microbiol..

[166] DeDiego M.L., Nieto-Torres J.L., Jimenez-Guardeno J.M., Regla-Nava J.A., Castano-Rodriguez C., Fernandez-Delgado R., Usera F., Enjuanes L. (2014). Coronavirus virulence genes with main focus on SARS-CoV envelope gene. Virus Res..

[167] Mesotten D., Meijs D.A.M., van Bussel B.C.T., Stessel B., Mehagnoul-Schipper J., Hana A., Scheeren C.I.E., Strauch U., van de Poll M.C.G., Ghossein-Doha C. (2021). Differences and Similarities Among Coronavirus Disease 2019 Patients Treated in Seven ICUs in Three Countries Within One Region: An Observational Cohort Study. Crit. Care Med..

[168] Asarnow D., Wang B., Lee W.H., Hu Y., Huang C.W., Faust B., Ng P.M.L., Ngoh E.Z.X., Bohn M., Bulkley D. (2021). Structural insight into SARS-CoV-2 neutralizing antibodies and modulation of syncytia. Cell.

[169] Lu Q., Liu J., Zhao S., Gomez Castro M.F., Laurent-Rolle M., Dong J., Ran X., Damani-Yokota P., Tang H., Karakousi T. (2021). SARS-CoV-2 exacerbates proinflammatory responses in myeloid cells through C-type lectin receptors and Tweety family member 2. Immunity.

[170] Savarino A., Boelaert J.R., Cassone A., Majori G., Cauda R. (2003). Effects of chloroquine on viral infections: An old drug against today’s diseases?. Lancet Infect. Dis..

[171] Ma L.L., Liu H.M., Liu X.M., Yuan X.Y., Xu C., Wang F., Lin J.Z., Xu R.C., Zhang D.K. (2021). Screening S protein—ACE2 blockers from natural products: Strategies and advances in the discovery of potential inhibitors of COVID-19. Eur. J. Med. Chem..

[172] Gies V., Bekaddour N., Dieudonne Y., Guffroy A., Frenger Q., Gros F., Rodero M.P., Herbeuval J.P., Korganow A.S. (2020). Beyond Anti-viral Effects of Chloroquine/Hydroxychloroquine. Front. Immunol..

[173] Ortiz M.E., Thurman A., Pezzulo A.A., Leidinger M.R., Klesney-Tait J.A., Karp P.H., Tan P., Wohlford-Lenane C., McCray P.B., Meyerholz D.K. (2020). Heterogeneous expression of the SARS-Coronavirus-2 receptor ACE2 in the human respiratory tract. EBioMedicine.

[174] Devaux C.A., Rolain J.M., Colson P., Raoult D. (2020). New insights on the antiviral effects of chloroquine against coronavirus: What to expect for COVID-19?. Int. J. Antimicrob. Agents.

[175] Wang N., Han S., Liu R., Meng L., He H., Zhang Y., Wang C., Lv Y., Wang J., Li X. (2020). Chloroquine and hydroxychloroquine as ACE2 blockers to inhibit viropexis of 2019-nCoV Spike pseudotyped virus. Phytomedicine.

[176] Yan Y., Zou Z., Sun Y., Li X., Xu K.F., Wei Y., Jin N., Jiang C. (2013). Anti-malaria drug chloroquine is highly effective in treating avian influenza A H5N1 virus infection in an animal model. Cell Res..

[177] Mitja O., Corbacho-Monne M., Ubals M., Alemany A., Suner C., Tebe C., Tobias A., Penafiel J., Ballana E., Perez C.A. (2021). A Cluster-Randomized Trial of Hydroxychloroquine for Prevention of COVID-19. N. Engl. J. Med..

[178] Fried M.W., Crawford J.M., Mospan A.R., Watkins S.E., Munoz B., Zink R.C., Elliott S., Burleson K., Landis C., Reddy K.R. (2021). Patient Characteristics and Outcomes of 11 721 Patients with Coronavirus Disease 2019 (COVID-19) Hospitalized Across the United States. Clin. Infect. Dis..

[179] Abella B.S., Jolkovsky E.L., Biney B.T., Uspal J.E., Hyman M.C., Frank I., Hensley S.E., Gill S., Vogl D.T., Maillard I. (2021). Efficacy and Safety of Hydroxychloroquine vs Placebo for Pre-exposure SARS-CoV-2 Prophylaxis Among Health Care Workers: A Randomized Clinical Trial. JAMA Intern. Med..

[180] Barnabas R.V., Brown E.R., Bershteyn A., Stankiewicz Karita H.C., Johnston C., Thorpe L.E., Kottkamp A., Neuzil K.M., Laufer M.K., Deming M. (2021). Hydroxychloroquine as Postexposure Prophylaxis to Prevent Severe Acute Respiratory Syndrome Coronavirus 2 Infection: A Randomized Trial. Ann. Intern. Med..

[181] Arabi Y.M., Gordon A.C., Derde L.P.G., Nichol A.D., Murthy S., Beidh F.A., Annane D., Swaidan L.A., Beane A., Beasley R. (2021). Lopinavir-ritonavir and hydroxychloroquine for critically ill patients with COVID-19: REMAP-CAP randomized controlled trial. Intensive Care Med..

[182] Magagnoli J., Narendran S., Pereira F., Cummings T., Hardin J.W., Sutton S.S., Ambati J. (2020). Outcomes of hydroxychloroquine usage in United States veterans hospitalized with COVID-19. medRxiv.

[183] Lebek S., Tafelmeier M., Messmann R., Provaznik Z., Schmid C., Maier L.S., Birner C., Arzt M., Wagner S. (2020). Angiotensin-converting enzyme inhibitor/angiotensin II receptor blocker treatment and haemodynamic factors are associated with increased cardiac mRNA expression of angiotensin-converting enzyme 2 in patients with cardiovascular disease. Eur. J. Heart Fail..

[184] Neyrinck A.P., Matthay M.A. (2009). The role of angiotensin-converting enzyme inhibition in endotoxin-induced lung injury in rats. Crit. Care Med..

[185] Melissa Hallow K., Dave I. (2021). RAAS Blockade and COVID-19: Mechanistic Modeling of Mas and AT1 Receptor Occupancy as Indicators of Pro-Inflammatory and Anti-Inflammatory Balance. Clin. Pharmacol. Ther..

[186] Yisireyili M., Uchida Y., Yamamoto K., Nakayama T., Cheng X.W., Matsushita T., Nakamura S., Murohara T., Takeshita K. (2018). Angiotensin receptor blocker irbesartan reduces stress-induced intestinal inflammation via AT1a signaling and ACE2-dependent mechanism in mice. Brain Behav. Immun..

[187] Hallaj S., Ghorbani A., Mousavi-Aghdas S.A., Mirza-Aghazadeh-Attari M., Sevbitov A., Hashemi V., Hallaj T., Jadidi-Niaragh F. (2021). Angiotensin-converting enzyme as a new immunologic target for the new SARS-CoV-2. Immunol. Cell Biol..

[188] Kriszta G., Kriszta Z., Vancsa S., Hegyi P.J., Frim L., Eross B., Hegyi P., Petho G., Pinter E. (2021). Effects of Angiotensin-Converting Enzyme Inhibitors and Angiotensin Receptor Blockers on Angiotensin-Converting Enzyme 2 Levels: A Comprehensive Analysis Based on Animal Studies. Front. Pharmacol..

[189] Zhang P., Zhu L., Cai J., Lei F., Qin J.J., Xie J., Liu Y.M., Zhao Y.C., Huang X., Lin L. (2020). Association of Inpatient Use of Angiotensin-Converting Enzyme Inhibitors and Angiotensin II Receptor Blockers with Mortality Among Patients with Hypertension Hospitalized with COVID-19. Circ. Res..

[190] Zhuang M.W., Cheng Y., Zhang J., Jiang X.M., Wang L., Deng J., Wang P.H. (2020). Increasing host cellular receptor-angiotensin-converting enzyme 2 expression by coronavirus may facilitate 2019-nCoV (or SARS-CoV-2) infection. J. Med. Virol..

[191] Mehta N., Kalra A., Nowacki A.S., Anjewierden S., Han Z., Bhat P., Carmona-Rubio A.E., Jacob M., Procop G.W., Harrington S. (2020). Association of Use of Angiotensin-Converting Enzyme Inhibitors and Angiotensin II Receptor Blockers with Testing Positive for Coronavirus Disease 2019 (COVID-19). JAMA Cardiol..

[192] Guzik T.J., Mohiddin S.A., Dimarco A., Patel V., Savvatis K., Marelli-Berg F.M., Madhur M.S., Tomaszewski M., Maffia P., D’Acquisto F. (2020). COVID-19 and the cardiovascular system: Implications for risk assessment, diagnosis, and treatment options. Cardiovasc. Res..

[193] Gross S., Jahn C., Cushman S., Bar C., Thum T. (2020). SARS-CoV-2 receptor ACE2-dependent implications on the cardiovascular system: From basic science to clinical implications. J. Mol. Cell Cardiol..

[194] Hemnes A.R., Rathinasabapathy A., Austin E.A., Brittain E.L., Carrier E.J., Chen X., Fessel J.P., Fike C.D., Fong P., Fortune N. (2018). A potential therapeutic role for angiotensin-converting enzyme 2 in human pulmonary arterial hypertension. Eur. Respir. J..

[195] Rey-Parra G.J., Vadivel A., Coltan L., Hall A., Eaton F., Schuster M., Loibner H., Penninger J.M., Kassiri Z., Oudit G.Y. (2012). Angiotensin converting enzyme 2 abrogates bleomycin-induced lung injury. J. Mol. Med..

[196] Hoepel W., Chen H.J., Geyer C.E., Allahverdiyeva S., Manz X.D., de Taeye S.W., Aman J., Mes L., Steenhuis M., Griffith G.R. (2021). High titers and low fucosylation of early human anti-SARS-CoV-2 IgG promote inflammation by alveolar macrophages. Sci. Transl. Med..

[197] Monteil V., Kwon H., Prado P., Hagelkruys A., Wimmer R.A., Stahl M., Leopoldi A., Garreta E., Hurtado Del Pozo C., Prosper F. (2020). Inhibition of SARS-CoV-2 Infections in Engineered Human Tissues Using Clinical-Grade Soluble Human ACE2. Cell.

[198] Huang K.Y., Lin M.S., Kuo T.C., Chen C.L., Lin C.C., Chou Y.C., Chao T.L., Pang Y.H., Kao H.C., Huang R.S. (2021). Humanized COVID-19 decoy antibody effectively blocks viral entry and prevents SARS-CoV-2 infection. EMBO Mol. Med..

[199] Zhang Y., Leung D.Y., Goleva E. (2014). Anti-inflammatory and corticosteroid-enhancing actions of vitamin D in monocytes of patients with steroid-resistant and those with steroid-sensitive asthma. J. Allergy Clin. Immunol..

[200] Bassatne A., Basbous M., Chakhtoura M., El Zein O., Rahme M., El-Hajj Fuleihan G. (2021). The link between COVID-19 and VItamin D (VIVID): A systematic review and meta-analysis. Metabolism.

[201] Peng M.Y., Liu W.C., Zheng J.Q., Lu C.L., Hou Y.C., Zheng C.M., Song J.Y., Lu K.C., Chao Y.C. (2021). Immunological Aspects of SARS-CoV-2 Infection and the Putative Beneficial Role of Vitamin-D. Int. J. Mol. Sci..

[202] Li Y.C., Qiao G., Uskokovic M., Xiang W., Zheng W., Kong J. (2004). Vitamin D: A negative endocrine regulator of the renin-angiotensin system and blood pressure. J. Steroid Biochem. Mol. Biol..

[203] Li Y.C., Kong J., Wei M., Chen Z.F., Liu S.Q., Cao L.P. (2002). 1,25-Dihydroxyvitamin D(3) is a negative endocrine regulator of the renin-angiotensin system. J. Clin. Investig..

[204] Xu S., Chen Y.H., Tan Z.X., Xie D.D., Zhang C., Xia M.Z., Wang H., Zhao H., Xu D.X., Yu D.X. (2015). Vitamin D3 pretreatment alleviates renal oxidative stress in lipopolysaccharide-induced acute kidney injury. J. Steroid Biochem. Mol. Biol..

[205] Mendonca P., Soliman K.F.A. (2020). Flavonoids Activation of the Transcription Factor Nrf2 as a Hypothesis Approach for the Prevention and Modulation of SARS-CoV-2 Infection Severity. Antioxidants.

